# Dacarbazine Combined Targeted Therapy versus Dacarbazine Alone in Patients with Malignant Melanoma: A Meta-Analysis

**DOI:** 10.1371/journal.pone.0111920

**Published:** 2014-12-11

**Authors:** Guan Jiang, Rong-Hua Li, Chao Sun, Yan-Qun Liu, Jun-Nian Zheng

**Affiliations:** 1 Department of Dermatology, Affiliated Hospital of Xuzhou Medical College, Xuzhou, 221002, China; 2 Jiangsu Key Laboratory of Biological Cancer Therapy, Xuzhou Medical College, Xuzhou, 221002, China; 3 Center for Disease Control and Prevention of Xuzhou City, Xuzhou, 221002, China; Shanghai Jiao Tong University School of Medicine, China

## Abstract

**Background:**

Malignant melanoma is the most aggressive and deadly form of skin cancer. Dacarbazine (DTIC) has been the approved first-line treatment for metastatic melanoma in routine clinical practice. However, response rates with single-agent DTIC are low. The objective of this study was to compare the efficacy and safety of DTIC with or without placebo and DTIC-based combination therapies in patients with advanced metastatic melanoma.

**Methods:**

We searched from electronic databases such as The Cochrane Library, MEDLINE, EBSCO, EMBASE, Ovid, CNKI, and CBMDisc from 2003 to 2013. The primary outcome measures were overall response and 1-year survival, and the secondary outcome measurements were adverse events.

**Results:**

Nine randomized controlled trials (RCTs) involving 2,481 patients were included in the meta-analysis. DTIC-based combination therapies was superior to DTIC alone in overall response (combined risk ratio [RR]  = 1.60, 95% confidence interval [CI]: 1.27–2.01) and 1-year survival (combined RR = 1.26, 95% CI: 1.14–1.39). Patients with DTIC-based combination therapies had higher incidence of adverse events including nausea (combined RR = 1.23, 95% CI: 1.10–1.36), vomiting (combined RR = 1.73, 95% CI: 1.41–2.12) and neutropenia (combined RR = 1.75, 95% CI: 1.42–2.16) compared to the group for DTIC alone.

**Conclusion:**

These data suggested that DTIC-based combination therapies could moderately improve the overall response and the 1-year survival but increased the incidence of adverse events. Further large-scale, high-quality, placebo-controlled, double-blind trials are needed to confirm this conclusion.

## Introduction

Malignant melanoma is the most aggressive form of skin cancer and is notoriously resistant to all current modalities of cancer therapy [1], [2]. Malignant melanoma accounts for only 4% of all dermatological malignancies, but is responsible for 80% of mortality from skin tumors [3]. Malignant melanoma is treated with a combination of therapies that include surgical removal, chemotherapy, and radiotherapy; however, the long-term survival of patients with malignant melanoma is not encouraging due to its chemoresistance and rapid metastasis [4]. The chemoresistance may be due to decreased drug uptake into cancer cells, increased drug efflux, intracellular drug inactivation, repair of drug-induced damage, or resistance to drug-induced apoptosis [5], [6]. A large set of genetic, functional and biochemical studies suggest that melanoma cells become “bullet-proof” against a variety of chemotherapeutic drugs [7].

Dacarbazine (DTIC) is the most active single agent for treatment of advanced metastatic melanoma and for more than 30 years has remained the standard chemotherapy for this malignancy [8]. DTIC methylates nucleic acids, causing DNA damage resulting in growth arrest and cell death. Unfortunately, the response rates for single-agent DTIC are disappointingly low, ranging from 10% to 20%, with complete responses seen in less than 5% of patients [9], [10]. Moreover, DTIC can cause gastrointestinal side effects such as nausea and vomiting, although most of them are mild and can be clinically manageable. It can also suppress the production of blood cells in the bone marrow, thereby causing anemia and neutropenia, and more rarely causes diarrhea and a flu-like syndrome 7 to 14 days after administration [11].

Therefore, new treatment approaches have been explored to improve therapeutic effectiveness against malignant melanoma while reducing side effects. A number of clinical studies regarding DTIC-based combination chemotherapy or biochemotherapy (with Interferon and/or Interleukin-2) showed slightly higher efficacy but didn't bring about more significant survival benefit while increasing side effects compared to DTIC alone [12]. In recent years, as molecular biology has developed rapidly and the mechanism of melanoma has been studied further, targeted therapy has made major breakthroughs. They include monoclonal antibody or inhibitors targeting to cell surface antigens or receptors, kinase inhibitors acting on cellular pathways, immune targeting drugs (such as anti-cytotoxic T lymphocyte-associated antigen-4 monoclonal antibody), anti-angiogenic drugs and BCL-2 antisense oligonucleotide drugs and so on. These drugs show good therapeutic potential, but there is no reliable evidence on whether the clinical response of DTIC could be enhanced by them. Consequently, it is necessary to comprehensively analyze the data from clinical RCTs to evaluate the efficacy and safety of DTIC alone versus DTIC combined targeted therapy in treatment of metastatic melanoma. In this article, we analyzed the results from eight recent RCTs for this purpose. These data may provide a basis for future clinical trial design.

## Methods

### Search strategy

We searched The Cochrane Library, MEDLINE, EBSCO, EMBASE, Ovid databases and clinical trial websites from 2003 to 2013. The search strategy included the keyword “DTIC” combined with the Medical Subject Headings (MeSH) “metastatic melanoma” and “randomized controlled trials”. We also searched Chinese databases such as CNKI and CBMDisc using the above search terms. The reference lists of all relevant articles were searched for further studies.

### Inclusion and exclusion criteria

The inclusion criteria included: (i) studies must be prospective randomized controlled clinical trials (RCTs); (ii) The subjects of the study must be diagnosed with advanced or metastatic melanoma by clinical pathology or cytology; (iii) they must include a single-agent DTIC (or with placebo) for the control group, and the comparison group (s) should be DTIC combined targeted therapy; (iv) the main outcome measures of literature must include overall response, 1-year survival and adverse events. The exclusion criteria were: (i) the research failed to provide the required information, such as the median overall survival time (mOS), overall response and 1-year survival, etc; (ii) repeated reports.

### Literature selection and data collection process

**Figure 1:**
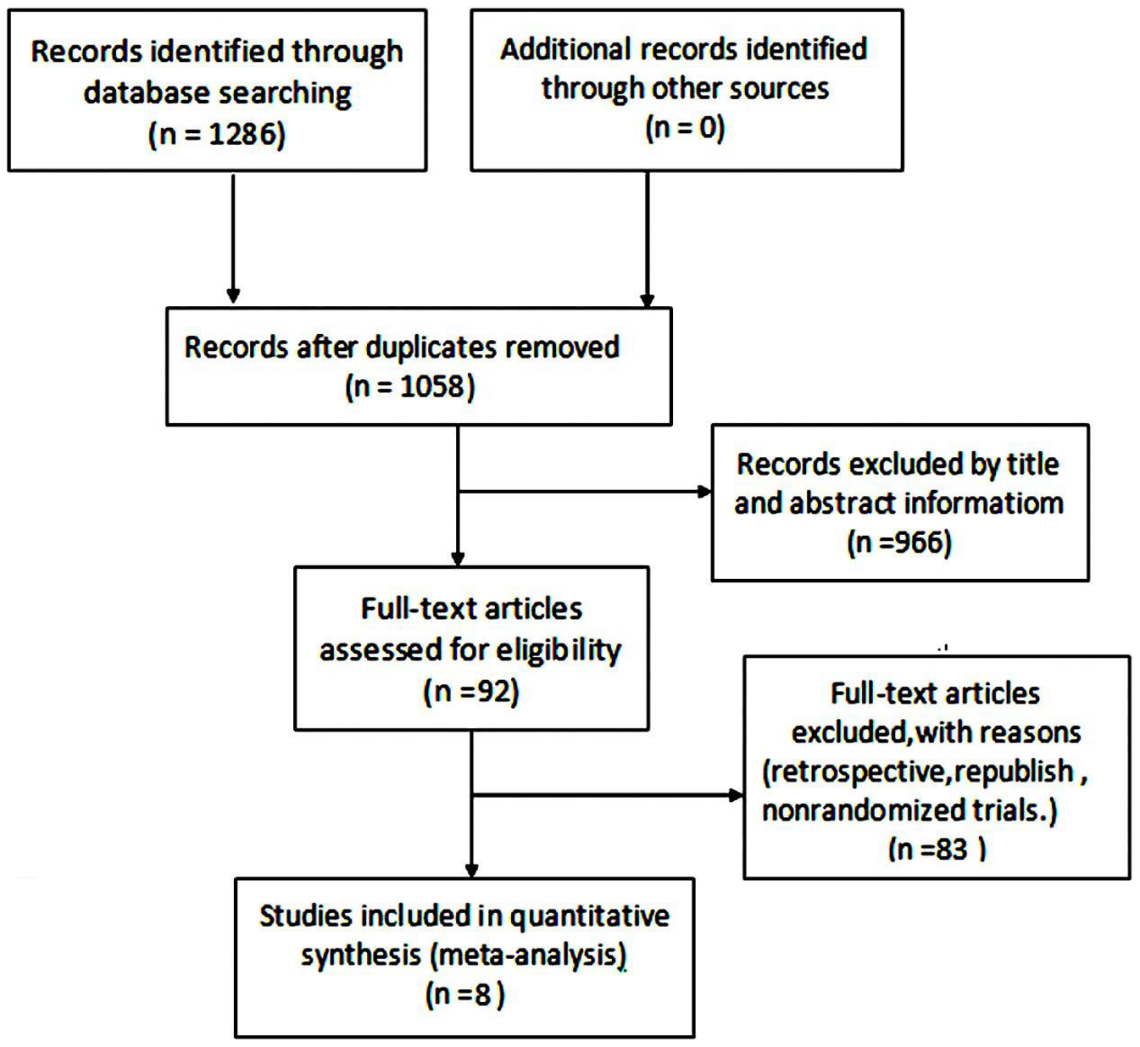
PRISMA flow chart of the meta-analysis.

Two investigators independently selected literature on the basis of the inclusion and exclusion criteria. The literature selection process is presented in the PRISMA flow chart (Fig. 1) according to the PRISMA guidelines [13], [14]. The two investigators then extracted data independently from the retrieved studies according to a standardized data extraction form that included patients, methods, interventions and outcomes. Disagreements were resolved by discussion among the investigators.

### Methodological quality assessment

**Figure 2:**
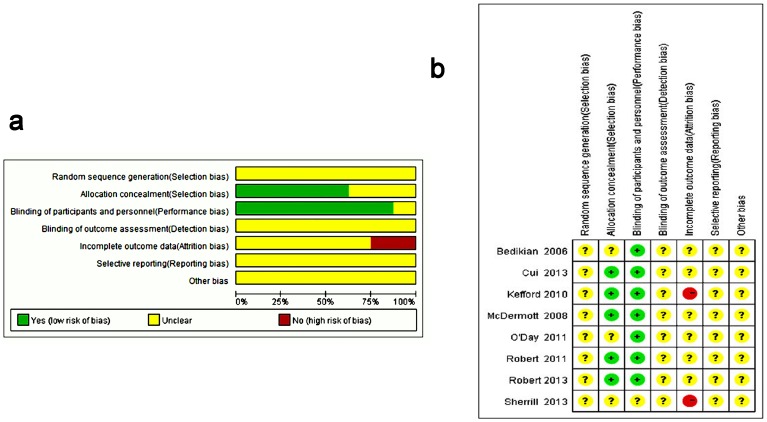
Risk of bias. (a) percentile chart and (b) summary diagram.

We evaluated the methodological quality of the included literature according to the RCT quality evaluation standard of the Cochrane Handbook for Systematic Reviews of Interventions, Version 5.0.0 (Fig. 2a). For each included study, two reviewers independently completed and assessed methodological quality.

### Definition of main outcomes

Complete response was defined as disappearance of all symptoms and signs of all measurable disease, lasting for at least 4 weeks, without appearance of new lesions. Partial response was defined as a >50% reduction in the sum of the products of the perpendicular diameters of all measurable lesions, lasting for at least 4 weeks, without the appearance of new lesions or enlargement of existing lesions. Overall response included complete response and partial response. Overall survival (OS) was defined as the time from the date of randomization to the date of death from any cause, or the date of last follow-up for living patients. So the 1-year survival was the proportion of participants alive at 1-year follow-up. As for the safety outcomes, we referred to the trial authors' definitions. In this meta-analysis, four frequent nonhematologic adverse events (nausea, vomiting, fatigue and constipation) and two frequent hematologic adverse events (anemia and neutropenia) were examined.

### Statistical methods

Meta-analysis was performed using Review Manager Version 5.0 software, which was provided by The Cochrane Collaboration [15]. Summary measures of efficacy and safety used relative risk (RR) for dichotomous variables, along with its 95% confidence interval (95% CI). The between-studies heterogeneity was evaluated using the Chi-square test, P values, and I^2^ statistics [16]. If there was no significant heterogeneity (P>0.1, I^2^<50%), the pooled RR was estimated by a fixed-effect model; if heterogeneity existed, we needed to analyze its sources and adopt subcategory analyses to factors that might contribute to the heterogeneity. If there was statistical heterogeneity among the studies without clinical heterogeneity or the difference was no clinical significance, a random-effects model was applied. If the heterogeneity between groups was too great, or sufficiently detailed data from the original trials were not available, a descriptive analysis could be adopted. Publication bias was estimated by funnel plots using Revman 5 software [17]. We also further examined the potential of publication bias using the Begg and Egger tests. In addition, sensitivity analysis was performed to examine the influence of individual studies. Begg tests, Egger tests and sensitivity analysis were performed with STATA version 12.

## Results

### Study selection

The initial search resulted in 1,286 potential citations, of which 228 duplicate papers were excluded. Of the remaining 1,058 articles, 966 were excluded after the title and abstract were read. Then, 92 full-text articles were assessed for eligibility. Eight possible candidates were retrieved for detailed examination by reading the full text [18]–[25]. The screening process is summarized in a flow diagram (Fig. 1).

### Study characteristics and quality assessment

The eight included studies, with a total of 2,221 participants, were all RCTs conducted between 2003 and 2013 that were available as fully published papers. The characteristics of the trials included are shown in Table 1. The eight included studies were two-group parallel-design studies, so eight comparisons were included this meta-analysis. Of all the included RCTs, none mentioned a specific randomization method, seven were blinded and five reported allocation concealment. Quality evaluations of the included studies are shown in Fig. 2.

**Table 1:** Summary of the characteristics of the included 8 trials.

Author Year	The tumor stage	Intervention(C/T)	Age (C/T)	No. of Patients	Dosage & duration
Bedikian et al. 2006. [18]	Stage III (unresectable) or stage IV melanoma	Dacarbazine	16–89	385	Dacarbazine (1,000 mg/m^2^) alone or preceded by a 5-day continuous intravenous infusion of oblimersen sodium (7 mg/kg/d) every 3 weeks for up to 8 cycles.
		Dacarbazine + Oblimersen sodium	17–93	386	
McDermott et al.2008. [19]	Stage III (unresectable) or stage IV melanoma	Placebo + Dacarbazine	18–88	50	On day 1 of a 21-day cycle, patients received intravenous dacarbazine 1,000 mg/m^2^ for a maximum of 16 cycles. Oral sorafenib 400 mg or placebo was administered twice a day continuously.
		Sorafenib + Dacarbazine	31–82	51	
Kefford et al. 2010. [20]	Stage IV melanoma	Placebo + Dacarbazine	58±14.8	40	Bosentan 500 mg twice daily or matching placebo, in addition to dacarbazine 1000 mg/m^2^ every 3 weeks.
		Bosentan + Dacarbazine	62.1±12.2	40	
Robert et al. 2013. [21]	Stage III and stage IV melanoma	Placebo + Dacarbazine	40–65	46	Oral selumetinib (75 mg twice daily in a 21-day cycle) or placebo; all patients received intravenous dacarbazine (1000 mg/m^2^ on day 1 of a 21-day cycle).
		Selumetinib + Dacarbazine	48–69	45	
Robert et al.2011. [22]	Stage III (unresectable) or stage IV melanoma	Placebo + Dacarbazine	56.4	252	Ipilimumab (10 mg/kg) plus dacarbazine (850 mg/m^2^) or dacarbazine (850 mg/m^2^) plus placebo, given at weeks 1, 4, 7, and 10, followed by dacarbazine alone every 3 weeks through week 23.
		Ipilimumab + Dacarbazine	57.5	250	
O'Day et al.2011. [23]	Stage IV melanoma	Placebo + Dacarbazine	56–74	32	1000 mg/m^2^ dacarbazine plus placebo,1000 mg/m^2^ dacarbazine plus 10 mg/kg intetumumab, each study agent once every 3 weeks for up to 8 cycles.
		Dacarbazine + Intetumumab	52–66	32	
Cui et al. 2013. [24]	Stage III (unresectable) or stage IV melanoma	Placebo + Dacarbazine	22–84	54	Dacarbazine 250 mg/m^2^ for up to a maximum of 12 cycles, on days 1–5 of a 21-day treatment cycle; Endostar (7.5 mg/m^2^) or placebo for up to a maximum of 12 cycles, once daily within 3–4 hours on days 1–14 of a 21-day treatment cycle.
		Endostar + Dacarbazine	17–76	56	
Sherrill et al. 2013. [25]	Stage III (unresectable) or stage IV melanoma	Placebo + Dacarbazine	None	252	Ipilimumab (10 mg/kg every 3 weeks ×4 doses, then every 12 weeks starting week 24) + DTIC (850 mg/m^2^ every 3 weeks ×8 doses); placebo (every 3 weeks ×4 doses, then every 12 weeks starting week 24) + DTIC (850 mg/m^2^ every 3 weeks ×8 doses).
		Ipilimumab + Dacarbazine	None	250	

Notes: T: Trial Group, C: Control Group.

### Effectiveness

Because all the included studies were RCTs, the RR was used as the effect size. Of those, six comparisons were used to analyze the overall response and eight comparisons to analyze the 1-year survival.

**Figure 3:**
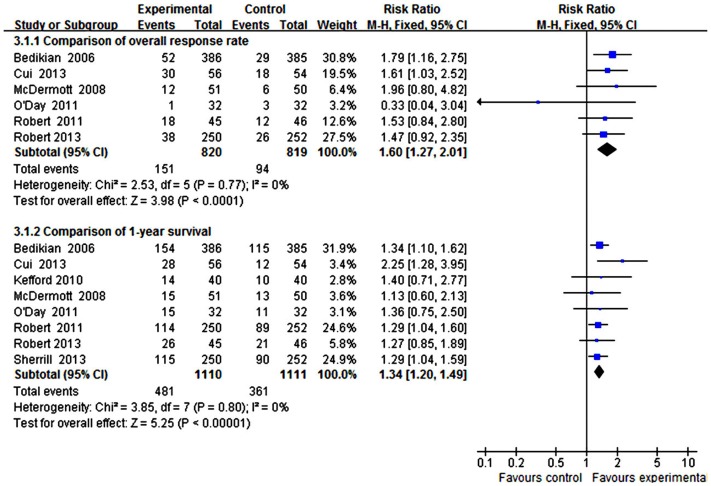
Forest plot of the efficacy of DTIC alone and DTIC combined targeted therapy.

**Figure 4:**
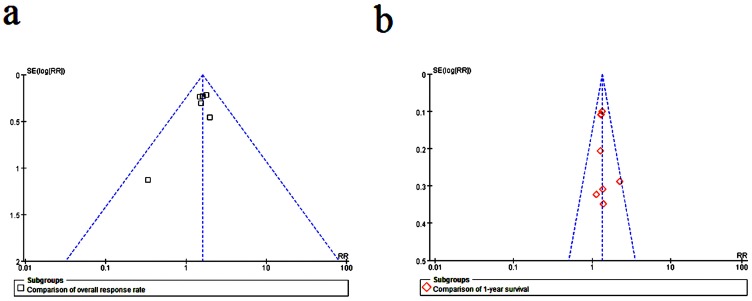
Funnel plot of the efficacy of DTIC alone and DTIC combined targeted therapy. (a) overall response rate and (b) 1-year survival.

For the overall response, there was no significant heterogeneity (I^2^ = 0%, P = 0.77); therefore, RR and 95% CI were calculated by a fixed-effects model. The overall response in the arm for DTIC combined targeted therapy was higher than that in the arm for DTIC alone (combined RR = 1.60; 95% CI, 1.27–2.01, Z = 3.98, P<0.0001) (Fig. 3). The corresponding funnel plot shows a symmetric distribution of studies, indicating no publication bias (Fig. 4a). Moreover, there was also no evidence of publication bias tested by constructing Begg's funnel plot or by Egger's test (P = 0.16) (see S1 appendix).

For the 1-year survival, there was no significant heterogeneity (I^2^ = 0%, P = 0.80) among the eight comparisons; therefore, RR and 95% CI were calculated by a fixed-effects model. The 1-year survival in the arm for DTIC combined targeted therapy was higher than that in the arm for DTIC alone (combined RR = 1.34; 95% CI, 1.20–1.49, Z = 5.25, P<0.00001) (Fig. 3). The corresponding funnel plot showed no publication bias (Fig. 4b). Moreover, there was no evidence of publication bias tested by constructing Begg's funnel plot or by Egger's test (P = 0.414) (see S2 appendix).

Furthermore, the sensitivity analysis was conducted for all the outcome measures of effectiveness. The results revealed that no individual study appeared to change the pooled RR dramatically (see S3 appendix for more detailed information).

### Safety

**Figure 5:**
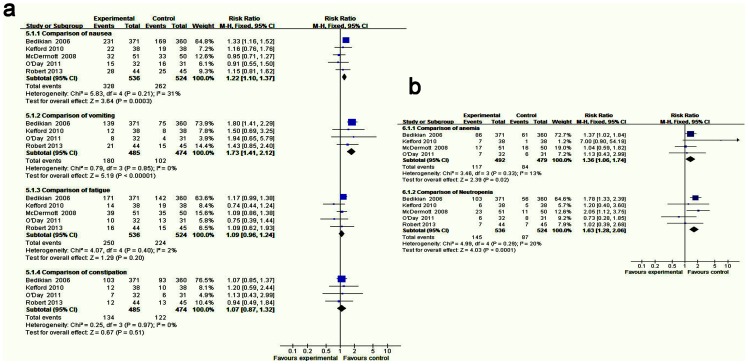
Forest plot of adverse events between DTIC alone and DTIC combined targeted therapy. (a) nonhematologic adverse events and (b) hematologic adverse events.

Nausea was reported in five trials. A fixed-effects model was used because there was no significant heterogeneity in these data sets: (I^2^ = 31%, P = 0.21). The result showed a significant difference between the arm for DTIC combined targeted therapy and the arm for DTIC alone (combined RR = 1.22, 95% CI: 1.10–1.37, Z = 3.64, P = 0.0003) (Fig. 5a).

Vomiting was reported in four trials. A fixed-effects model was used because there was no significant heterogeneity (I^2^ = 0%, P = 0.85). The result showed a significant difference between the arm for DTIC combined targeted therapy and the arm for DTIC alone (combined RR = 1.73, 95% CI: 1.41–2.12, Z = 5.19, P<0.00001) (Fig. 5a).

Fatigue was reported in five trials. A fixed-effects model was used because there was no significant heterogeneity (I^2^ = 2%, P = 0.40). We failed to show a significant difference between the arm for DTIC combined targeted therapy and the arm for DTIC alone (combined RR = 1.09, 95% CI: 0.96–1.24, Z = 1.29, P = 0.20) (Fig. 5a).

Constipation was reported in four trials. A fixed-effects model was used because there was no significant heterogeneity (I^2^ = 0%, P = 0.97). We failed to show a significant difference between the arm for DTIC combined targeted therapy and the arm for DTIC alone (combined RR = 1.07, 95% CI: 0.87–1.32, Z = 0.67, P = 0.51) (Fig. 5a).

Anemia was reported in four trials. A fixed-effects model was used because there was no significant heterogeneity (I^2^ = 13%, P = 0.33). It showed that there was a significant difference between the arm for DTIC combined targeted therapy and the arm for DTIC alone (combined RR = 1.36, 95% CI: 1.06–1.74, Z = 2.39, P = 0.02) (Fig. 5b).

Neutropenia was reported in five trials. A fixed-effects model was used because there was no significant heterogeneity (I^2^ = 20%, P = 0.29). It showed there was a significant difference between the arm for DTIC combined targeted therapy and the arm for DTIC alone (combined RR = 1.63, 95% CI: 1.28–2.06, Z = 4.03, P<0.0001) (Fig. 5b).

Furthermore, sensitivity analysis was conducted for all safety outcome measures. The results revealed that the study conducted by Bedikian and his co-worker changed the pooled RR dramatically (see We found that except for vomiting there were no significant differences in these safety outcomes between the arm for DTIC combined targeted therapy and the arm for DTIC alone. In another subgroup, there was only one study, conducted by Bedikian and his co-worker, in which we found significant differences between the arm for DTIC combined targeted therapy and the arm for DTIC alone for adverse events other than fatigue and constipation.

## Discussion

Malignant melanoma is a malignant tumor of neural crest origin [24]. It is formed from malignant melanocytes located at the bottom of the epidermis and is almost always evolved from a mole or pigmented spot. Melanoma is curable surgically when discovered at early stages; however, once regional and systemic spread of the disease occurs, treatment options are limited and are generally considered ineffective [26], [27]. The median overall survival is poor, averaging 6 to 9 months [28]. DTIC is the most commonly used therapy for metastatic melanoma, with a median progression-free survival (PFS) of 1.5 to 1.6 months and no improvement in OS [17]. Compared with DTIC alone, most DTIC-based combination chemotherapy or biochemotherapy (with IFN and/or IL-2) have yielded poor improvements with respect to either PFS or OS. In 2001, Huncharek et al. [29] conducted a meta-analysis of 20 RCTs including 3,273 patients with stage IV malignant melanoma, and the results showed that the response rate for the combination therapy of DTIC plus interferon-α was 53%, which was greater than that for DTIC alone, but no significant difference was observed in OS.

In 2011, two agents, ipilimumab (a fully human monoclonal antibody that blocks CTLA-4 to promote antitumor immunity) and vemurafenib (a potent inhibitor of mutated V600E BRAF) were approved in Europe and the US for the treatment of metastatic melanoma. Compared with DTIC alone, ipilimumab in combination with DTIC has been shown to improve OS in an RCT in patients with previously treated metastatic melanoma, while vemurafenib improved OS and PFS in an RCT in patients with previously untreated melanoma harboring the V600 BRAF mutation [22], [30]. In addition, sorafenib (an inhibitor of Raf kinase) and Endostar (a potent novel endogenous angiogenic inhibitor) can also improve the efficacy of DTIC in patients with advanced melanoma. More and more studies have demonstrated that DTIC combined targeted therapy can significantly improve the PFS and OS of patients with metastatic melanoma, so it is necessary to comprehensively analyze the data from clinical RCTs to evaluate the efficacy and safety of DTIC alone versus DTIC combined targeted therapy in treatment of metastatic melanoma.

In this meta-analysis, the result showed that the group for DTIC combined targeted therapy was superior to the group for DTIC alone in overall response rate (combined RR = 1.60, 95% CI: 1.27–2.01) and 1-year survival rate (combined RR = 1.34, 95% CI: 1.20–1.49). In addition, in terms of safety analysis, we found that DTIC combined targeted therapy had no higher incidence of most adverse events (including nausea, fatigue, constipation, anemia and neutropenia but excluding vomiting) compared with DTIC alone. However, oblimersen sodium (BCL-2 antisense oligonucleotide drugs) in combination with DTIC had a higher incidence of adverse events (including nausea, vomiting, anemia and neutropenia) compared with DTIC alone.

In summary, the available evidence shows that DTIC combined targeted therapy may moderately improve the overall response and the 1-year survival, although it may increase the incidence of some adverse events.

## Supporting Information

S1 Appendix Begg's Test and Egger's test of the overall response rate of DTIC alone and DTIC combined targeted therapy.
(DOCX)

S2 Appendix Begg's Test and Egger's test of the 1-year survival of DTIC alone and DTIC combined targeted therapy.
(DOCX)

S3 Appendix Sensitivity analysis (with or without subgroup analyzes) of the efficacy and safety of DTIC alone and DTIC combined targeted therapy. (1) Overall response rate; (2) 1-year survival; (3) Nausea; (4) Vomiting; (5) Fatigue; (6) Constipation; (7) Anemia; (8) Neutropenia.
(DOCX)

S1 Checklist PRISMA 2009 Checklist.
(DOC)
