# Supplementary material for: Dacarbazine Combined Targeted Therapy versus Dacarbazine Alone in Patients with Malignant Melanoma: A Meta-Analysis
Source: PLoS One. 2014 Dec 11;9(12):e111920. doi: 10.1371/journal.pone.0111920 (PMC4263472; doi:10.1371/journal.pone.0111920)

**Begg’s Test and Egger’s test of the overall response rate of DTIC alone and DTIC combined targeted therapy**


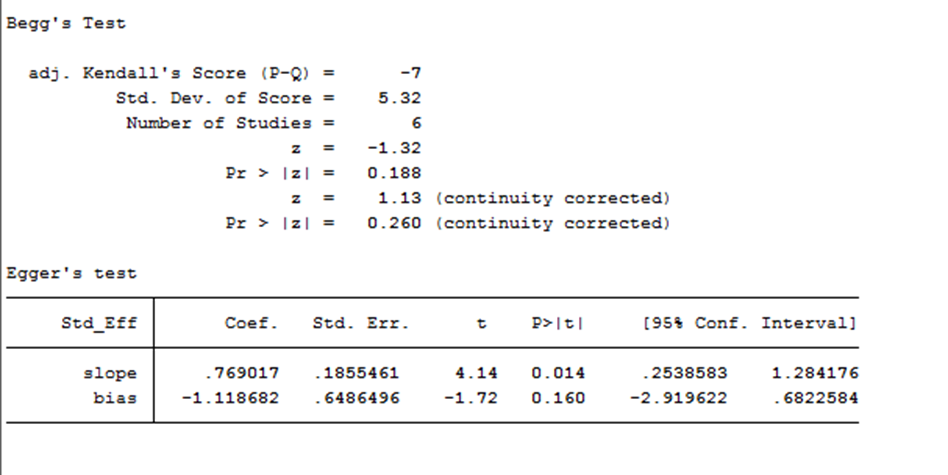


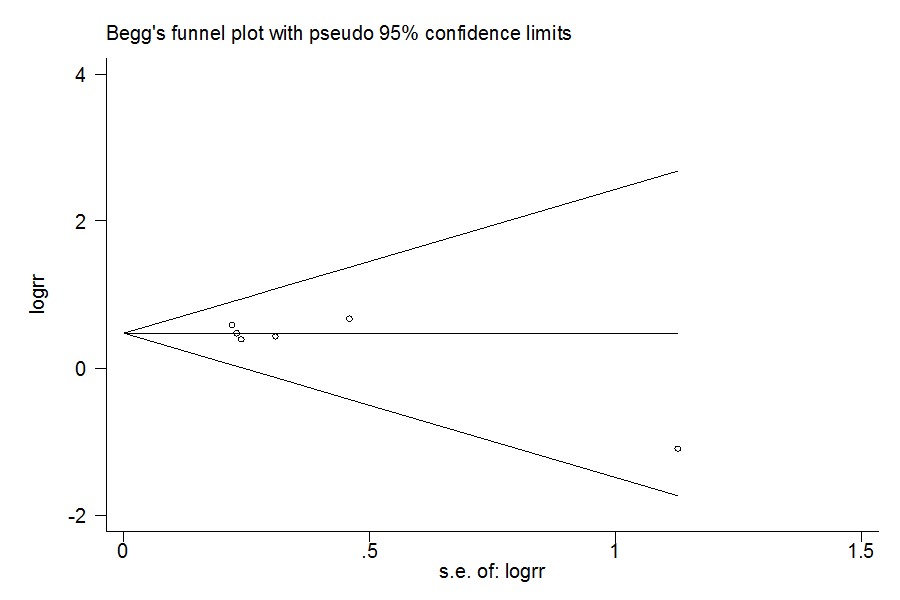

Supplement: S1 Appendix — Begg's Test and Egger's test of the overall response rate of DTIC alone and DTIC combined targeted therapy. (DOCX) [file pone.0111920.s001.docx]
