# Supplementary material for: Dacarbazine Combined Targeted Therapy versus Dacarbazine Alone in Patients with Malignant Melanoma: A Meta-Analysis
Source: PLoS One. 2014 Dec 11;9(12):e111920. doi: 10.1371/journal.pone.0111920 (PMC4263472; doi:10.1371/journal.pone.0111920)

**Sensitivity analysis（with or without subgroup analyzes）of the efficacy and safety of DTIC alone and DTIC combined targeted therapy** (A group is the subgroup of which the study conducted by Bedikian and his co-worker was removed out , B subgroup contains only one study conducted by Bedikian and his co-worker)

1. **Overall response rate**


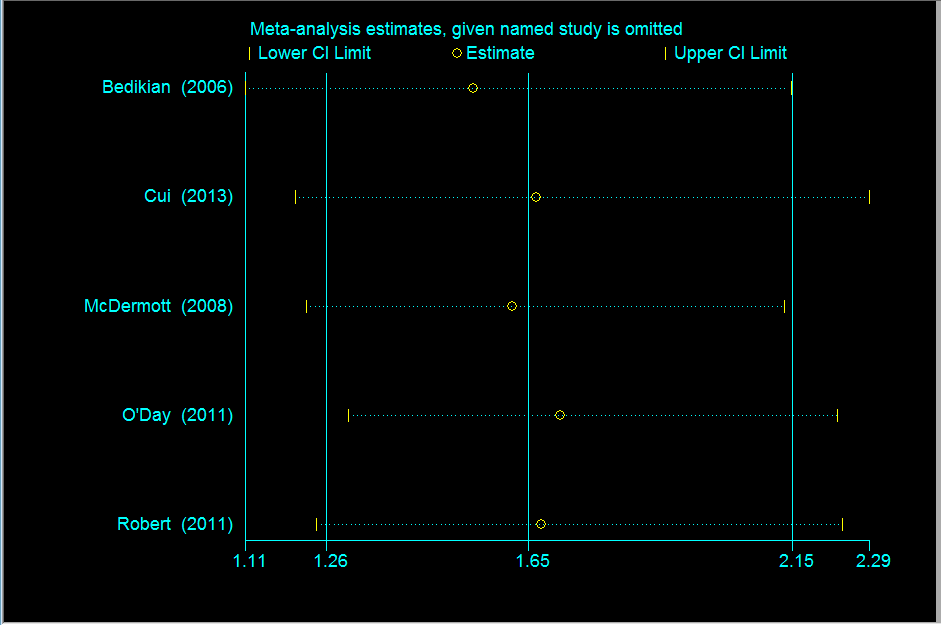


1. **1-year survival**


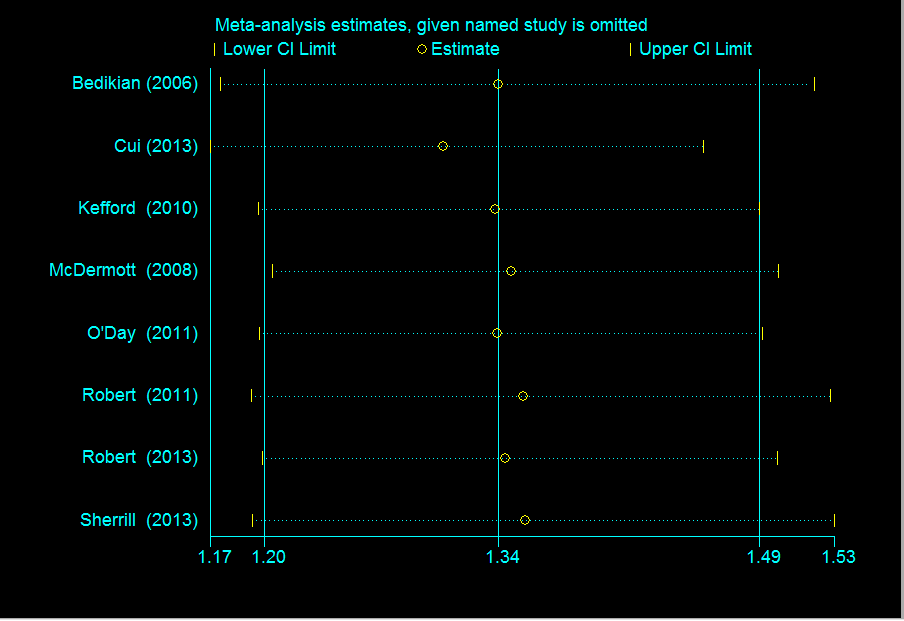


1. **Nausea**


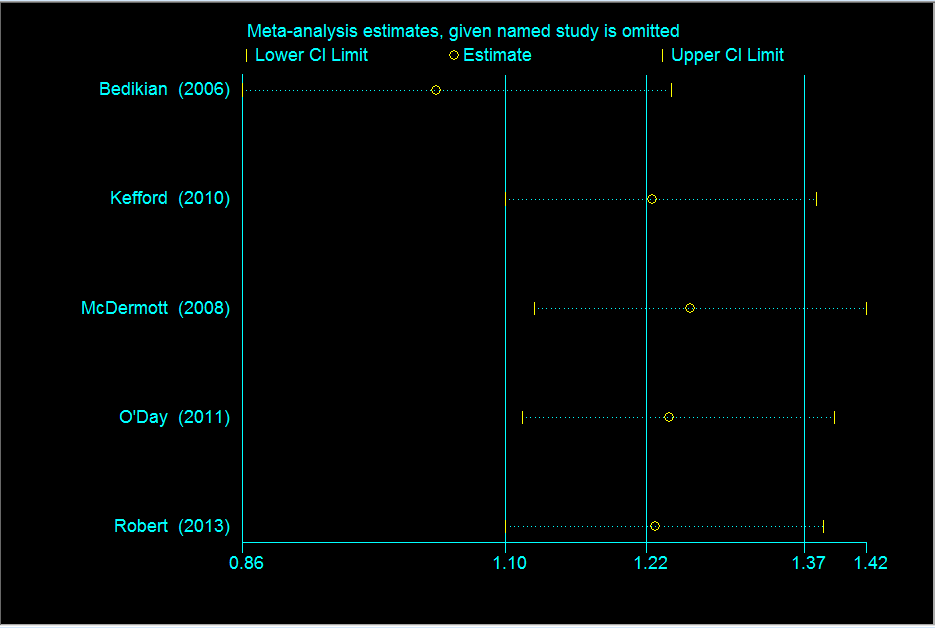


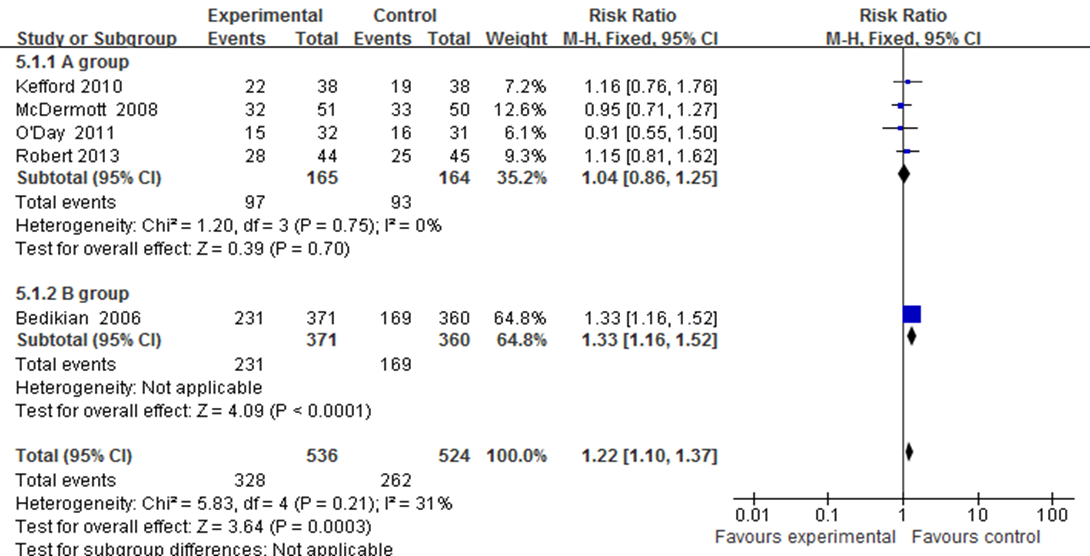


1. **Vomiting**


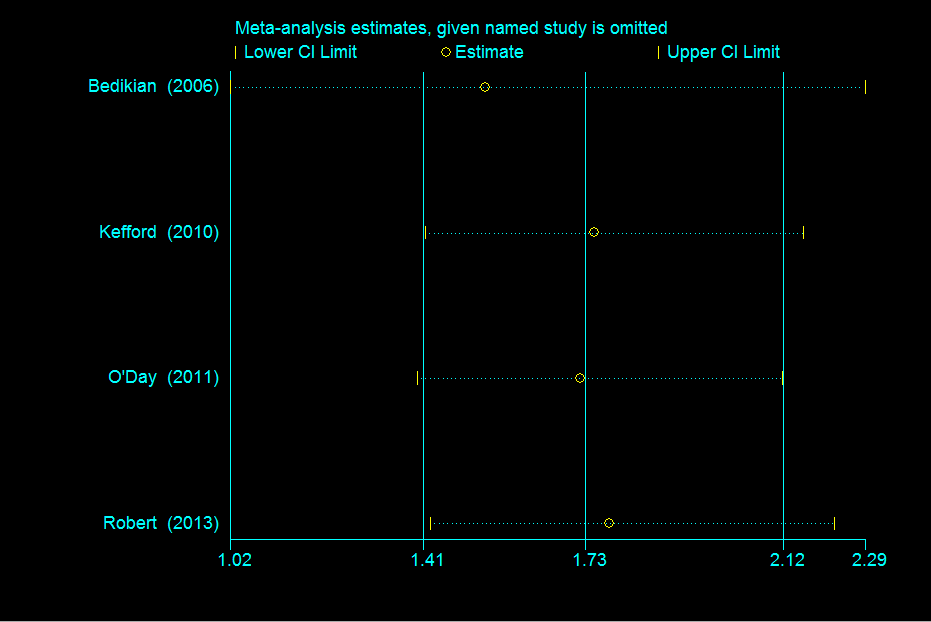


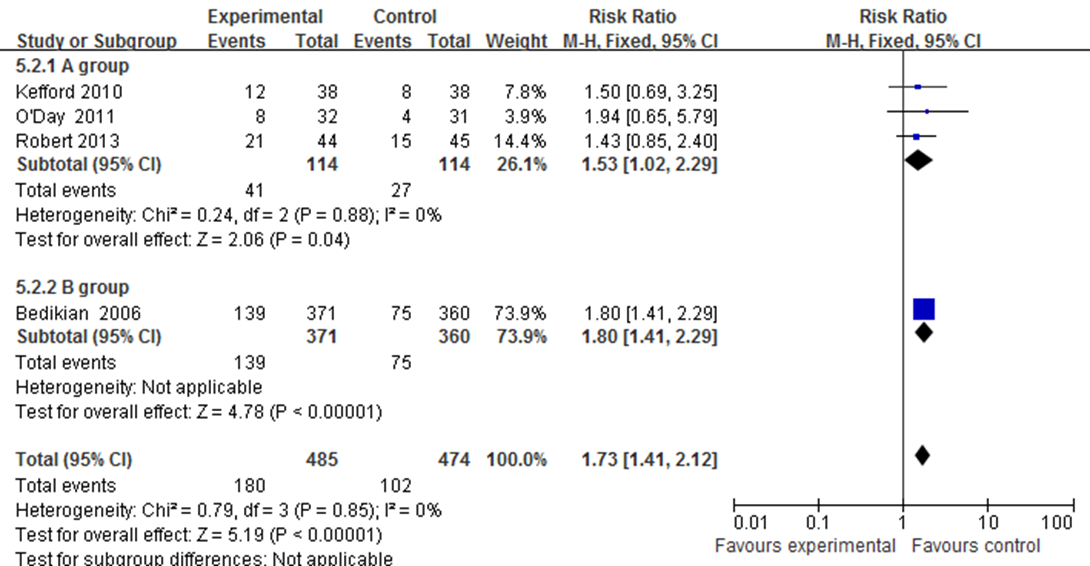


1. **Fatigue**


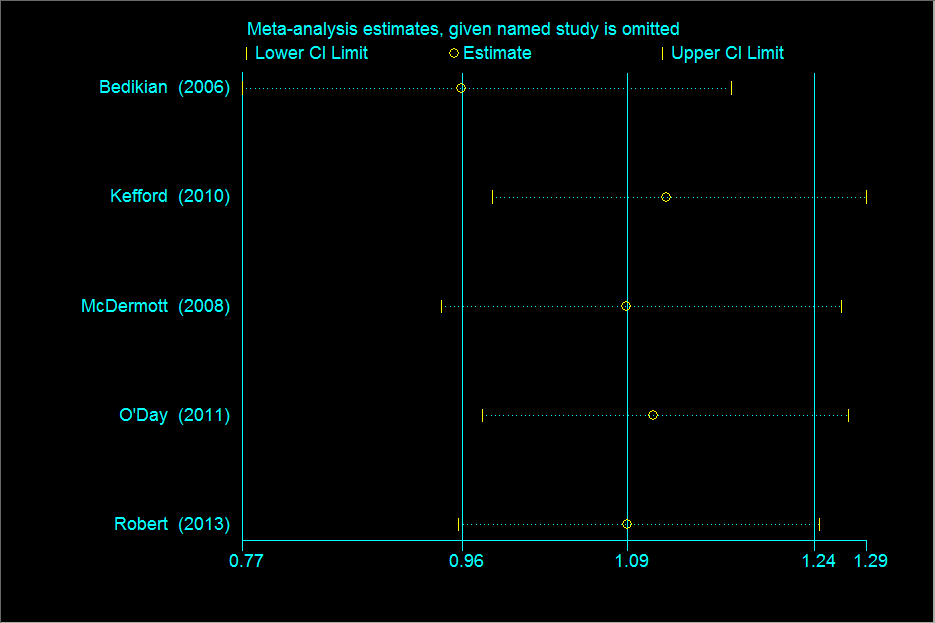


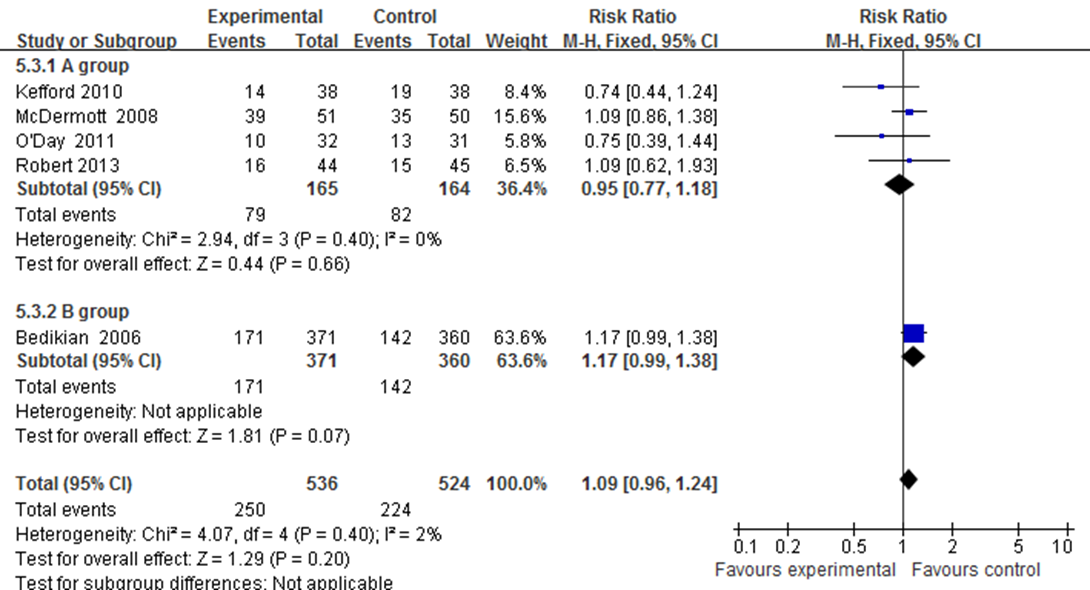


1. **Constipation**


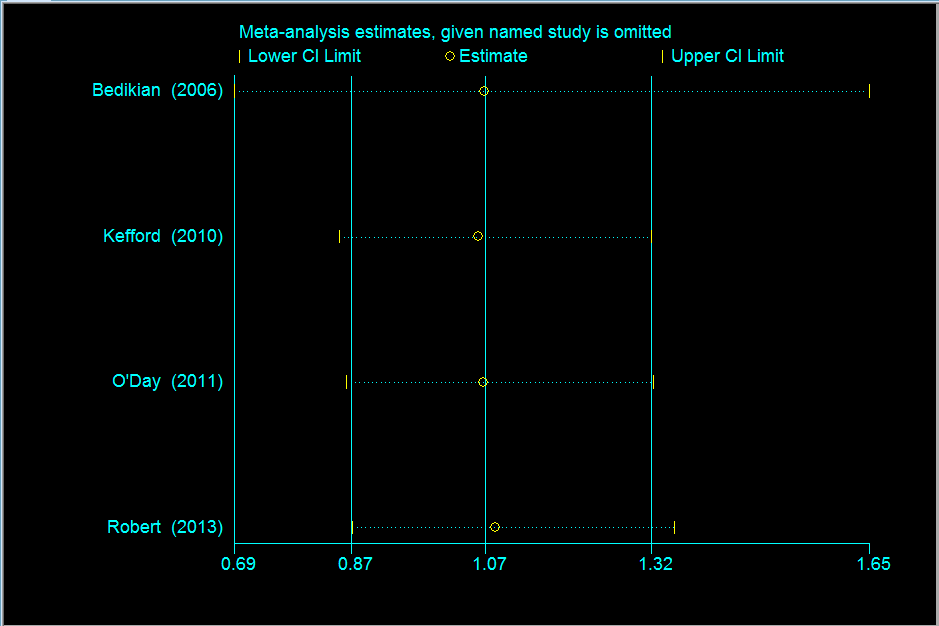


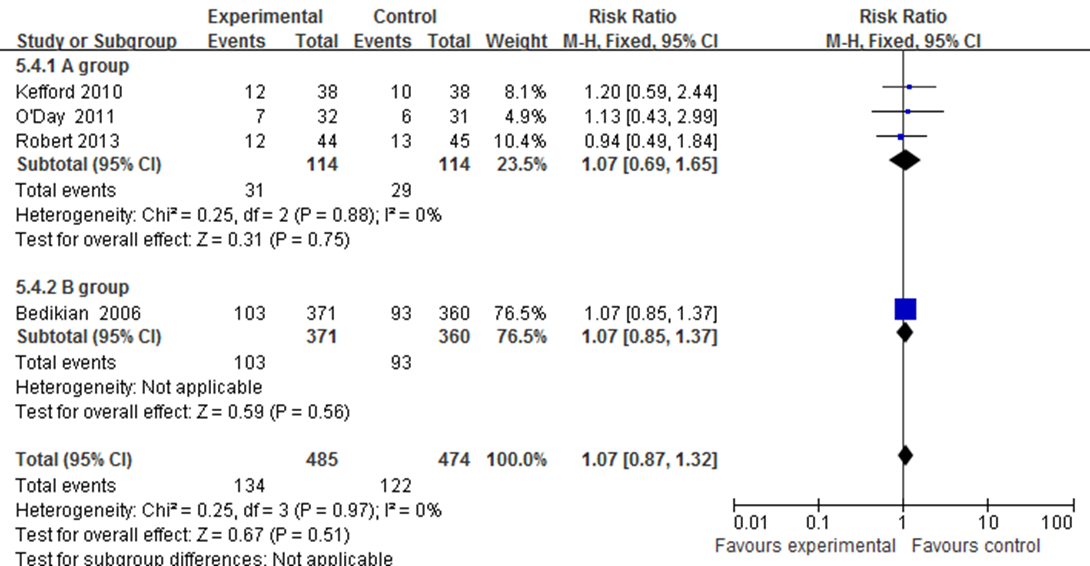


1. **Anemia**


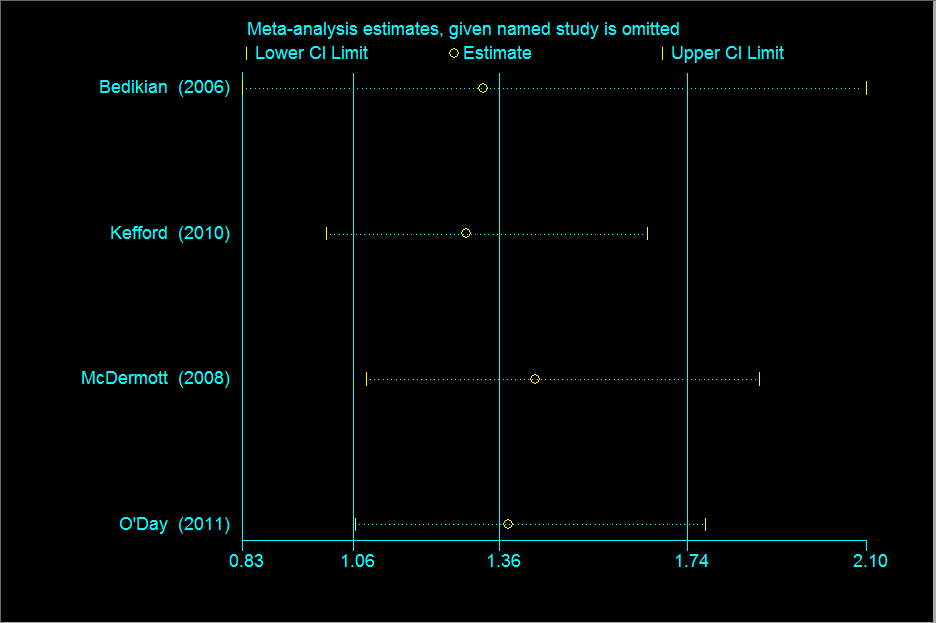


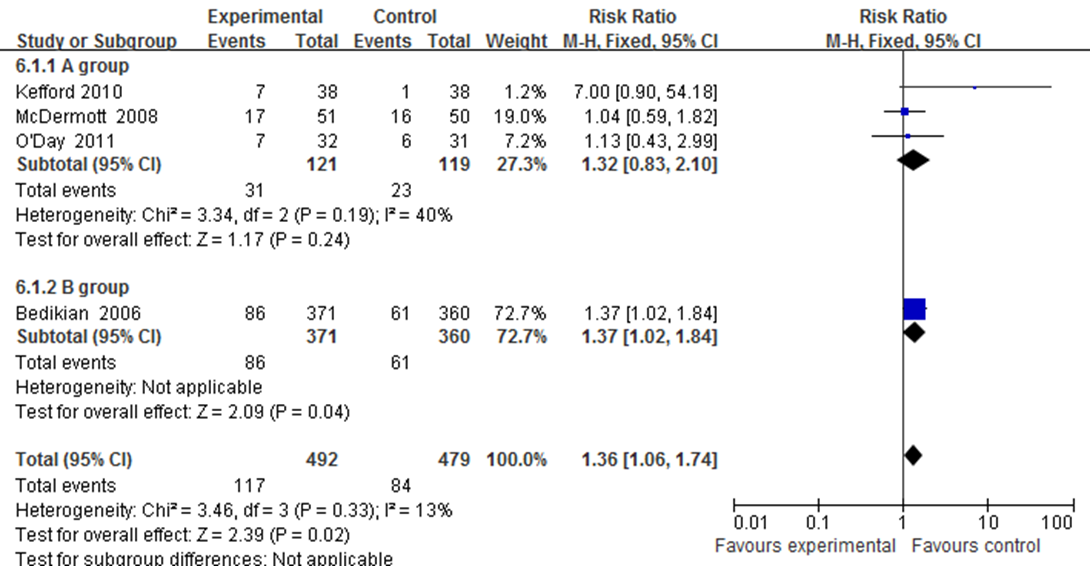


1. **Neutropenia**


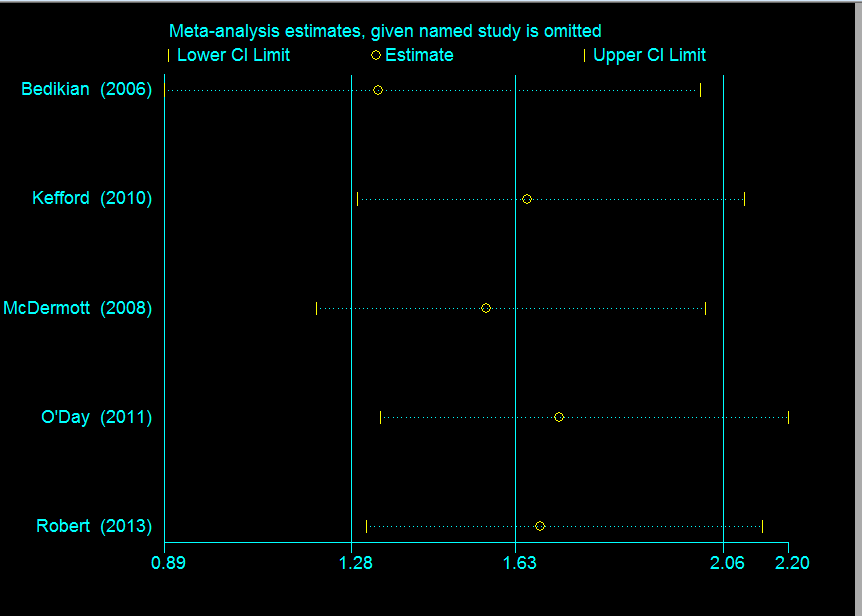


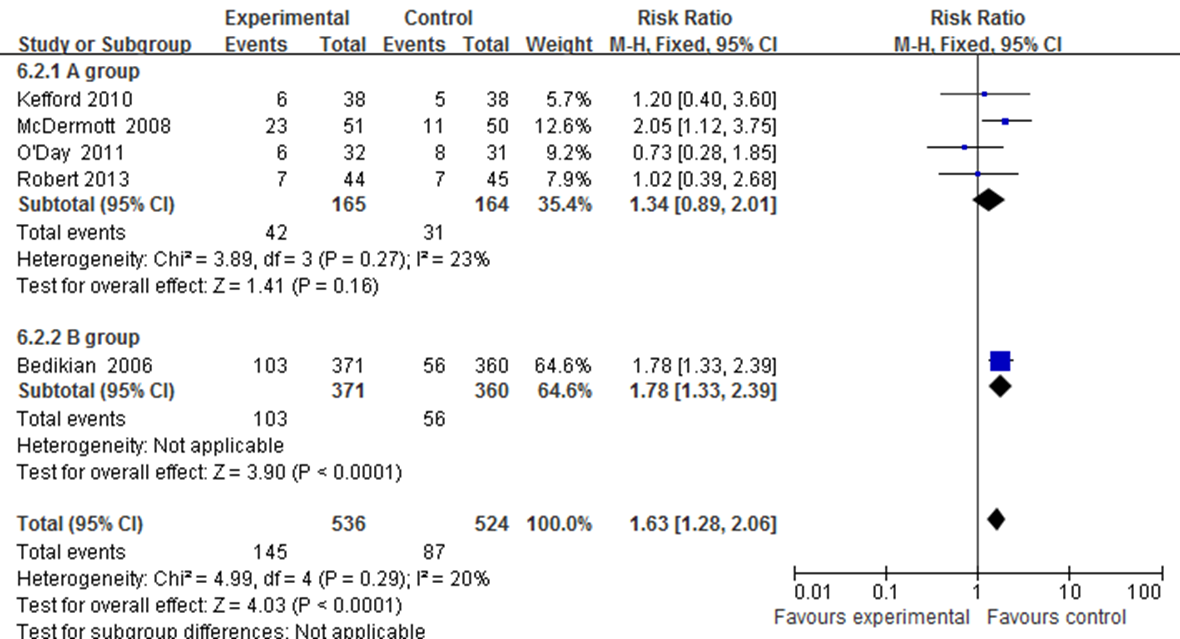

Supplement: S3 Appendix — Sensitivity analysis (with or without subgroup analyzes) of the efficacy and safety of DTIC alone and DTIC combined targeted therapy. (1) Overall response rate; (2) 1-year survival; (3) Nausea; (4) Vomiting; (5) Fatigue; (6) Constipation; (7) Anemia; (8) Neutropenia. (DOCX) [file pone.0111920.s003.docx]
