# Supplementary material for: Dacarbazine Combined Targeted Therapy versus Dacarbazine Alone in Patients with Malignant Melanoma: A Meta-Analysis
Source: PLoS One. 2014 Dec 11;9(12):e111920. doi: 10.1371/journal.pone.0111920 (PMC4263472; doi:10.1371/journal.pone.0111920)
Supplement: S1 Checklist — PRISMA 2009 Checklist. (DOC) [file pone.0111920.s004.doc]

| **Section/topic** | **#** | **Checklist item** | **Reported on page #** |
| --- | --- | --- | --- |
| **TITLE** | | |  |
| Title | 1 | Identify the report as a systematic review, meta-analysis, or both.  **Meta-analysis**  **Title: Dacarbazine-based chemotherapy or chemoimmunotherapy versus dacarbazine alone in patients with malignant melanoma: a meta-analysis from 9 Randomized Controlled Clinical Trials.** | 1 |
| **ABSTRACT** | | |  |
| Structured summary | 2 | Provide a structured summary including, as applicable: background; objectives; data sources; study eligibility criteria, participants, and interventions; study appraisal and synthesis methods; results; limitations; conclusions and implications of key findings; systematic review registration number.  **Background:** Malignant melanoma is the most aggressive and deadly form of skin cancer and its worldwide incidence is increasing rapidly every year. Dacarbazine (DTIC) has been the approved first-line treatment for metastatic melanoma in routine clinical practice. However, response rates with single-agent DTIC are low. This meta-analysis aims to compare the efficacy and safety of DTIC with or without placebo and DTIC-based chemotherapy or chemoimmunotherapy in patients with advanced metastatic melanoma.  **Methods:** We searched electronic databases such as the Cochrane library, MEDLINE，EBSCO，EMBASE，Ovid, cNKI, and cBMDisc from 2003-2013**.** The primary outcome measures were overall response rate and 1-year survival rate, and the secondary outcome measurements were adverse events..  **Results:** There were 9 randomized controlled trials (RCTs) involving 2481 patients enrolled in the meta-analysis. DTIC-based chemotherapy or chemoimmunotherapy was superior to DTIC in overall response rate and 1-year survival rate, with the combined risk ratio (RR) value and 95% confidence interval (CI) 1.60 (1.27~2.01) and 1.26(1.14~1.39), respectively. Patients with DTIC-based chemotherapy or chemoimmunotherapy had higher incidence of adverse events including nausea, vomiting and neutropenia compared to the DTIC monotherapy group. The combined RR value and 95% CI were 1.23 (1.10~1.36), 1.73 (1.41~2.12) and 1.75 (1.42~2.16), respectively.  **Conclusion:** These data suggested that DTIC-based chemotherapy or chemoimmunotherapy could moderately improve the overall response rate and the 1-year survival rate, but increased the incidence of adverse events. Future more large-scale, high-quality, placebo-controlled, double-blind trials are needed to confirm this conclusion. | 1-3 |
| **INTRODUCTION** | | |  |
| Rationale | 3 | Describe the rationale for the review in the context of what is already known.  At present, there is indeed no reliable evidence to prove the positive effect of DTIC-based chemotherapy or chemoimmunotherapy in the treatment of metastatic melanoma. Consequently, it is necessary to comprehensively analyze the data from clinical RCTs and evaluate the efficacy and safety of DTIC alone and DTIC-based chemotherapy or chemoimmunotherapy on metastatic melanoma. | 3- 4 |
| Objectives | 4 | Provide an explicit statement of questions being addressed with reference to participants, interventions, comparisons, outcomes, and study design (PICOS).  We evaluated the efficacy and safety of DTIC alone compared to DTIC-based chemotherapy or chemoimmunotherapy in the treatment of metastatic melanoma from recent clinical randomized controlled trials | 4 |
| **METHODS** | | |  |
| Protocol and registration | 5 | Indicate if a review protocol exists, if and where it can be accessed (e.g., Web address), and, if available, provide registration information including registration number.  **Note: The PICOS structure is not applicable to this paper.** |  |
| Eligibility criteria | 6 | Specify study characteristics (e.g., PICOS, length of follow-up) and report characteristics (e.g., years considered, language, publication status) used as criteria for eligibility, giving rationale.  The inclusion criteria included: (i) studies must be the prospective clinical randomized controlled trials (RCTs) of 2003-2013; (ii) the languages were not restricted; (iii)they must include a single-agent DTIC (or with placebo) for the control group, and the comparison group(s) should be DTIC-based chemotherapy or chemoimmunotherapy; (iv) the research must be for the treatment of malignant melanoma; (v) The main outcome measures of literature included overall response rate, 1-year survival rate and adverse events. | 5 |
| Information sources | 7 | Describe all information sources (e.g., databases with dates of coverage, contact with study authors to identify additional studies) in the search and date last searched.  We searched electronic databases such as the Cochrane library, MEDLINE，EBSCO，EMBASE，Ovid, cNKI, and cBMDisc from 2003-2013**.** | 4 |
| Search | 8 | Present full electronic search strategy for at least one database, including any limits used, such that it could be repeated.  We searched the Cochrane library, MEDLINE，EBSCO，EMBASE, Ovid databases and clinical trial websites from 2003 to 2013. The search strategy included keyword “DTIC” combined with the Medical Subject Headings (MeSH) “metastatic melanoma” and “randomized controlled trials”. we also searched the Chinese databases such as cNKI and cBMDisc using the above search terms. The reference lists of all relevant articles were searched for further studies. No language restriction was applied. | 5 |
| Study selection | 9 | State the process for selecting studies (i.e., screening, eligibility, included in systematic review, and, if applicable, included in the meta-analysis).  Two investigators independently selected literature on the basis of “Inclusion and exclusion criteria”, and the literature selection course was present in the PRISMA flow chart according to the PRISMA guidelines [12], [13]. | 5-6 |
| Data collection process | 10 | Describe method of data extraction from reports (e.g., piloted forms, independently, in duplicate) and any processes for obtaining and confirming data from investigators.  Two investigators extracted data independently from retrieved studies according to a standardized data extraction form, including patients, methods, interventions and outcomes. Disagreements were resolved by discussion among the investigators. | 6 |
| Data items | 11 | List and define all variables for which data were sought (e.g., PICOS, funding sources) and any assumptions and simplifications made.  **Note: The PICOS structure is not applicable to this paper.** |  |
| Risk of bias in individual studies | 12 | Describe methods used for assessing risk of bias of individual studies (including specification of whether this was done at the study or outcome level), and how this information is to be used in any data synthesis.  We evaluated the methodological quality of the included literature，according to RCT quality evaluation standard of Cochrane review manual 5.0. as follows: (1) Random sequence generation (selection bias)；(2) Allocation concealment(selection bias); (3) Blinding of participants and personnel(performance bias); (4) Blinding of outcome assessment(detection bias); (5) Incomplete outcome data(attrition bias); (6) Selective reporting(reporting bias); (7) Other bias. For each included study, two reviewers independently completed and assessed methodological quality. | 6 |
| Summary measures | 13 | State the principal summary measures (e.g., risk ratio, difference in means).  the relative risks (RR) | 7 |
| Synthesis of results | 14 | Describe the methods of handling data and combining results of studies, if done, including measures of consistency (e.g., I^2^) for each meta-analysis.  Meta-analysis was performed using the Review Manager Version 5.0 software, which was provided by the Cochrane Collaboration. The between-studies heterogeneity was evaluated using the Chi-square test, P values, and I^2^ statistics. There was a significant heterogeneity when P<0.1, I^2^>50％. | 7-8 |

Page 1 of 2

| **Section/topic** | **#** | **Checklist item** | **Reported on page #** |
| --- | --- | --- | --- |
| Risk of bias across studies | 15 | Specify any assessment of risk of bias that may affect the cumulative evidence (e.g., publication bias, selective reporting within studies).  Estimation of publication bias was shown by funnel plot. | 8 |
| Additional analyses | 16 | Describe methods of additional analyses (e.g., sensitivity or subgroup analyses, meta-regression), if done, indicating which were pre-specified.  **Note: The PICOS structure is not applicable to this paper.** |  |
| **RESULTS** | | |  |
| Study selection | 17 | Give numbers of studies screened, assessed for eligibility, and included in the review, with reasons for exclusions at each stage, ideally with a flow diagram.  The initial search resulted in 1286 potential citations, of which 228 repetition literatures were excluded. Of the remaining 1058 articles, 966 were excluded after reading title and abstract. Then, 92 full-text articles assessed for eligibility. 9 possible candidates were retrieved for detailed examination by reading the full text. The screening process is summarized in a flow diagram (Figure 1). | Page 8,  Figure 1 |
| Study characteristics | 18 | For each study, present characteristics for which data were extracted (e.g., study size, PICOS, follow-up period) and provide the citations.  The 9 studies included, a total of 2481 participants, were all randomized clinical trials of 2003 - 2013, which were available as fully published papers. The characteristics of the trials included are shown in Table 1. Among the 9 included studies, one was four-group design study, while the remaining 8 were two-group parallel design studies. | Page 8,  Table 1 |
| Risk of bias within studies | 19 | Present data on risk of bias of each study and, if available, any outcome level assessment (see item 12).  Of all the included RCTs, none mentioned specific random method, 7 were blinded and 5 reported allocation concealment. The quality evaluations of the included study are shown in Figure 2. | Page 8,  Figure 2 |
| Results of individual studies | 20 | For all outcomes considered (benefits or harms), present, for each study: (a) simple summary data for each intervention group (b) effect estimates and confidence intervals, ideally with a forest plot.  See the forest plot. | Figure 3,5,6 |
| Synthesis of results | 21 | Present results of each meta-analysis done, including confidence intervals and measures of consistency.  Overall response rate: heterogeneity (I^2^=0％，P=0.77), the combined RR value was 1.60 [(95% CI, 1.27~2.01), Z = 3.98, P<0.0001]; 1-year survival rate: heterogeneity (I^2^=25％，P=0.20), The combined RR value was 1.26 [(95%CI, 1.14-1.39), Z=4.49, P<0.00001]; Nausea: heterogeneity (I^2^=15%, P=0.31), the combined RR value 1.23 [(95% CI: 1.10-1.36), Z=3.78, P=0.0002]; Vomiting: heterogeneity (I^2^=0%, P=0.85), the combined RR value of 1.73 [(95% CI: 1.41-2.12), Z=5.19, P<0.00001]; Fatigue: heterogeneity (I^2^=2%, P=0.40), the combined RR value 1.09 [(95% CI: 0.96~1.24), Z=1.29, P=0.20]; Constipation : heterogeneity (I2=0%, P=0.97), the combined RR value 1.07 [(95% CI: 0.87~1.32), Z=0.67, P=0.51];Anemia: heterogeneity (I^2^=45%, P=0.09), the combined RR value 1.66 [(95% CI: 1.00~2.75), Z=1.98, P=0.05]; Neutropenia: heterogeneity (I^2^=28%, P=0.20), the combined RR value 1.75 [(95% CI: 1.42~2.16), Z=5.24, P<0.0001]. | 9-11 |
| Risk of bias across studies | 22 | Present results of any assessment of risk of bias across studies (see Item 15).  See the Risk of bias percentile chart and Risk of bias Summary diagram, and publication bias of the corresponding funnel plot. | Figure 2,  Figure 4 |
| Additional analysis | 23 | Give results of additional analyses, if done (e.g., sensitivity or subgroup analyses, meta-regression [see Item 16]).  **Note: The PICOS structure is not applicable to this paper.** |  |
| **DISCUSSION** | | |  |
| Summary of evidence | 24 | Summarize the main findings including the strength of evidence for each main outcome; consider their relevance to key groups (e.g., healthcare providers, users, and policy makers).  The result showed that the group DTIC-based chemotherapy or chemoimmunotherapy was superior to group DTIC in overall response rate and 1-year survival rate with the combined RR value and 95% CI, respectively, 1.60 (1.27~2.01) and 1.26 (1.14~1.39). In addition, in terms of safety analysis, we found that the incidence of nausea, vomiting and neutropenia in DTIC-based chemotherapy or chemoimmunotherapy of metastatic melanoma was higher than that in DTIC alone, but the incidence of fatigue, constipation and anemia was not significantly different in the two groups. According to our analysis, DTIC-based chemotherapy or chemoimmunotherapy had higher the incidence of adverse events and serious adverse events compared with DTIC alone group, but most adverse events were clinically manageable and there were few patients discontinuation of treatment because of adverse events. | 13 |
| Limitations | 25 | Discuss limitations at study and outcome level (e.g., risk of bias), and at review-level (e.g., incomplete retrieval of identified research, reporting bias).  In this study, we chose randomized controlled trials that are mostly connected with two samples. Our results indicate that the two approaches may have synergistic effect in the treatment of metastatic melanoma. However, the synergistic mechanism between the two therapeutic approaches remains to be illustrated. Moreover, we only selected a small number of RCTs, thus it is difficult to perform subgroup or sensitivity analyses. | 14 |
| Conclusions | 26 | Provide a general interpretation of the results in the context of other evidence, and implications for future research.  In summary, the available evidence shows that the DTIC-based chemotherapy or chemoimmunotherapy may moderately improve the response rate and the 1-year survival rate with increasing the incidence of adverse events, but most adverse events were clinically manageable. Therefore, DTIC-based chemotherapy or chemoimmunotherapy should be recommended in clinical practice but non serious adverse events must be monitored carefully. | 14 |
| **FUNDING** | | |  |
| Funding | 27 | Describe sources of funding for the systematic review and other support (e.g., supply of data); role of funders for the systematic review.  This project is supported by Grants from the National Natural Science Foundation of China (No. 81372916), and the Science and Technology Department of Xuzhou city (No. XM13B084). | 14 |

*From:*  Moher D, Liberati A, Tetzlaff J, Altman DG, The PRISMA Group (2009). Preferred Reporting Items for Systematic Reviews and Meta-Analyses: The PRISMA Statement. PLoS Med 6(6): e1000097. doi:10.1371/journal.pmed1000097

For more information, visit: **www.prisma-statement.org**.

Page 2 of 2
